# A Comprehensive Evaluation of GeneLEAD VIII DNA Platform Combined to Deeplex Myc-TB^®^ Assay to Detect in 8 Days Drug Resistance to 13 Antituberculous Drugs and Transmission of *Mycobacterium tuberculosis* Complex Directly From Clinical Samples

**DOI:** 10.3389/fcimb.2021.707244

**Published:** 2021-10-29

**Authors:** Isabelle Bonnet, Vincent Enouf, Florence Morel, Vichita Ok, Jérémy Jaffré, Vincent Jarlier, Alexandra Aubry, Jérôme Robert, Wladimir Sougakoff

**Affiliations:** ^1^ Assistance Publique–Hôpitaux de Paris (AP-HP), Hôpital Pitié-Salpêtrière, Service de Bactériologie-Hygiène, Paris, France; ^2^ Centre National de Référence des Mycobactéries et de la Résistance des Mycobactéries aux Antituberculeux (CNR-MyRMA), Paris, France; ^3^ Sorbonne Université, INSERM, Centre d’Immunologie et des Maladies Infectieuses (CIMI-Paris), Unité Mixte de Recherche (UMR) 1135, Paris, France; ^4^ Plateforme de Microbiologie Mutualisée (P2M), Pasteur International Bioresources network (PIBnet), Institut Pasteur, Paris, France

**Keywords:** GeneLEAD VIII, Deeplex Myc-TB, *Mycobacterium tuberculosis*, diagnostic, resistance, spoligotype

## Abstract

The GeneLEAD VIII (Diagenode, Belgium) is a new, fully automated, sample-to-result precision instrument for the extraction of DNA and PCR detection of *Mycobacterium tuberculosis* complex (MTBC) directly from clinical samples. The Deeplex Myc-TB^®^ assay (Genoscreen, France) is a diagnostic kit based on the deep sequencing of a 24-plexed amplicon mix allowing simultaneously the detection of resistance to 13 antituberculous (antiTB) drugs and the determination of spoligotype. We evaluated the performance of a strategy combining the both mentioned tools to detect directly from clinical samples, in 8 days, MTBC and its resistance to 13 antiTB drugs, and identify potential transmission of strains from patient-to-patient. Using this approach, we screened 112 clinical samples (65 smear-negative) and 94 MTBC cultured strains. The sensitivity and the specificity of the GeneLEAD/Deeplex Myc-TB approach for MTBC detection were 79.3% and 100%, respectively. One hundred forty successful Deeplex Myc-TB results were obtained for 46 clinical samples and 94 strains, a total of 85.4% of which had a Deeplex Myc-TB susceptibility and resistance prediction consistent with phenotypic drug susceptibility testing (DST). Importantly, the Deeplex Myc-TB assay was able to detect 100% of the multidrug-resistant (MDR) MTBC tested. The lowest concordance rates were for pyrazinamide, ethambutol, streptomycin, and ethionamide (84.5%, 81.5%, 73%, and 55%, respectively) for which the determination of susceptibility or resistance is generally difficult with current tools. One of the main difficulties of Deeplex Myc-TB is to interpret the non-synonymous uncharacterized variants that can represent up to 30% of the detected single nucleotide variants. We observed a good level of concordance between Deeplex Myc-TB-spoligotyping and MIRU-VNTR despite a lower discriminatory power for spoligotyping. The median time to obtain complete results from clinical samples was 8 days (IQR 7–13) provided a high-throughput NGS sequencing platform was available. Our results highlight that the GeneLEAD/Deeplex Myc-TB approach could be a breakthrough in rapid diagnosis of MDR TB in routine practice.

## Introduction

In 2019, tuberculosis (TB) was responsible for more deaths than any other infectious disease in the world (Furin et al., 2019). According to the World Health Organization (WHO), an estimated number of 10 million people have contracted TB and approximately 1.5 million died from the disease in 2019 (World Health Organization, 2020). In order to rapidly implement appropriate treatment against potentially resistant strains, drug-sensitive or drug-resistant TB must be quickly and accurately identified to avoid the selection of additional resistance, improve treatment outcomes, and reduce transmissibility (World Health Organization, 2018a; World Health Organization, 2018b; World Health Organization, 2019; Nahid et al., 2019). Because *Mycobacterium tuberculosis* complex (MTBC) is a slow-growing bacterium [average delay of 30 days between culture and phenotypic drug susceptibility testing (DST)], genotypic DST, especially from specimens, appears to be more advantageous than phenotypic DST.

Among the genotypic DST methods available to date, the widely used Xpert^®^ MTB/RIF test (Cepheid, USA) is an automated molecular unit test that identifies MTBC but only predicts rifampin resistance in clinical specimens (Boehme et al., 2010). Semi-automated tests such as the Fluorotype^®^ MTB test (Hain, Germany) combined with the Anyplex™ II MTB/MDR and MTB/XDR kits (Seegene, Korea) have been designed for large runs and are capable of detecting both MTBC and resistance to rifampicin, isoniazid, fluoroquinolones, and injectable drugs (Hofmann-Thiel and Hoffmann, 2014; Igarashi et al., 2017). Nevertheless, misidentifications, particularly with regard to resistance to rifampicin and fluoroquinolones, have been reported in the literature (Igarashi et al., 2017). DNA strip testing such as the MTBDRplus and MTBDRsl tests (Hain, Germany), and Sanger sequencing of genes involved in drug resistance can also detect drug resistance mutations (Brossier et al., 2009; Brossier et al., 2016; Brossier et al., 2017). However, both methods remain technically time-consuming, expensive, complex, and generally need to be performed on cultures to give good results. Recently, the development of next-generation sequencing (NGS) has led to widespread use of whole-genome sequencing (WGS) for TB diagnosis, drug resistance detection, and MTBC typing (Colman et al., 2019; Meehan et al., 2019; Nimmo et al., 2019; van Beek et al., 2019). However, some progress remains to be made in terms of the timeliness of results, performance when applied directly on clinical samples/data interpretation even if large systematic reviews have been published, and standardization to implement WGS in the routine clinical setting (Votintseva et al., 2017; Doyle et al., 2018; Meehan et al., 2019; World Health Organization, 2021).

Here, we report the combination of a new all-in-one automated molecular diagnostic platform performing DNA extraction and PCR amplification of MTBC (GeneLEAD VIII, Diagenode, Belgium) with a PCR-NGS-based all-in-one test for species identification, genotyping, and prediction of resistance to antituberculous (antiTB) drugs (Deeplex Myc-TB, Genoscreen, France) (Feuerriegel et al., 2021; Jouet et al., 2021; Kambli et al., 2021). The GeneLEAD/Deeplex Myc-TB approach has been compared with routine genotypic and phenotypic methods. Applied to samples or cultures, the Deeplex Myc-TB assay can provide identification, genotyping, and prediction of resistance to 13 antiTB drugs with a median delay of 8 days. To our knowledge, this is the first report of this combined approach on collected clinical samples.

## Materials and Methods

### Clinical Samples

One hundred twelve clinical samples of various types were prospectively included in the study between November 2018 and November 2019 (see **Table 1**). They were received from French clinical laboratories for suspicion of resistant TB in the frame of our activity as Mycobacteria National Reference Centre (Myc-NRC) or for TB diagnostic from different wards of our hospital. The samples were stored at 4°C when they could not be processed the day of reception. Each sample was submitted to auramine staining for microscopic examination and cultured on Löwenstein–Jensen medium using standard procedures and biosecurity recommendations (European Centre for Disease Prevention and Control, 2018).

**Table 1:** Strains and clinical samples.

Nature of clinical samples	*N*
Sputum	33
Gastric aspirate	6
Bronchial aspirate	37
Bronchoalveolar lavage	4
Lung biopsy	4
Pleural fluid	3
Lymph node	12
Spine	3
Abscess of the iliac fossa	1
Cerebrospinal fluid	6
Subdural hematoma	1
Skin sample	2
*Total*	*112*
**Microscopic examination of clinical samples**	
≥100 AFB/field	10
10 to 99 AFB/field	18
1 to 9 AFB/field	12
<1 AFB/field >10/slide	7
Negative	65
*Total*	*112*
**Strains**	
Liquid	39
Solid	55
*Total*	*94*

### Strains

In order to better evaluate the performance of the strategy in detecting mutations responsible for drug resistance, 94 MTBC strains were also included in the study. The panel included 189 unique mutations potentially involved in resistance to rifampicin (RIF, *n* = 19), isoniazid (INH, *n* = 10), ethionamide (ETH, *n* = 24), pyrazinamide (PZA, *n* = 39), ethambutol (EMB, *n* = 19), fluoroquinolones (FQ, *n* = 17), injectable drugs including kanamycin (KAN), amikacin (AMK), and capreomycin (CAP) (*n* = 11), streptomycin (STR, *n* = 40), linezolid (LNZ, *n* = 2), and bedaquiline (BDQ, *n* = 8). These strains were received on solid (*n* = 55) or liquid (*n* = 39) media from French laboratories between November 2018 and November 2019, for confirmation of resistance and additional susceptibility tests. The strains were stored at room temperature when they could not be processed the day of reception. Moreover, six frozen DNA sent to the Myc-NRC for suspicion of resistant TB between November 2018 and November 2019 were tested. Three were obtained from samples and three from cultures. DNA extraction was performed in the shipper’s laboratories.

### DNA Extraction

DNA extraction was performed using the GeneLEAD VIII system (Diagenode, Belgium) for clinical samples. Briefly, the principle of GeneLEAD is based on the DNA adsorption to magnetic particles after cell lysis; the DNA is recovered after a washing step followed by an elution. The required sample volume is 200 µl and the elution volume may be 50, 100, or 200 µl. Heat shock extraction was used for positive cultures and strains, as previously described (Brossier et al., 2010).

### GeneLEAD VIII Platform

The platform was used with the R-DiaMTBComplex™ real-time PCR detection kit (Diagenode, Belgium). DNA extracted by GeneLEAD VIII system from clinical samples was directly used for Deeplex Myc-TB analysis.

### Deeplex Myc-TB

The 24-plex PCR (Genoscreen, France) was then carried out according to the manufacturer’s recommendations and the PCR products were sent at Institut Pasteur of Paris to be sequenced on the the Mutualized Platform for Microbiology (P2M) using the Nextera XT DNA Library Preparation kit (Illumina Inc.) and the NextSeq 500 sequencing system (Illumina Inc.). The raw data were uploaded and analyzed on a secure cloud platform with an automated analysis pipeline with integrated reference databases. For each MTBC isolate, the results of 18 main gene targets known to be involved in MTBC resistance to first- and second-line drugs were generated: *rpoB*, *inhA*, *fabG1*, *katG*, *ahpC*, *pncA*, *embB*, *gidB*, *rpsL*, *rrs*, *eis*, *tlyA*, *gyrA*, *gyrB*, *ethA*, *rrl*, *rplC*, and *rv0678*. The average depth of coverage (x) and the reference coverage (%) were worked out online by the GenoScreen pipeline for each resistance gene. Based on previously observed mutations at these loci and interrogation of the available databases, the strains were ranked as susceptible, resistant, or “undetermined resistant” to each antibiotic (Miotto et al., 2014; Walker et al., 2015; Feuerriegel et al., 2015; Miotto et al., 2017). In addition to antibiotic resistance prediction, MTBC strains were characterized at the species level with the *hsp65* gene, at the genotype level with phylogenetic SNPs, and at the spoligotype level with the presence–absence pattern of 43 direct repeats at the CRISPR locus (Kamerbeek et al., 1997; Dale et al., 2001).

### Routine DST Procedures

The approach evaluated in the present study was compared to routine genotypic and phenotypic DST. The genetic variations involved in drug resistance were assessed by the line probe assays (MTBDRplus v2 and MTBDRsl v1, Hain Lifescience, Germany) or in-house PCR combined with Sanger sequencing of the genes responsible for antiTB drug resistance, as previously reported (Brossier et al., 2017). Each MTBC isolate was typed using mycobacterial interspersed repetitive unit variable-number tandem repeat (MIRU-VNTR) (Supply et al., 2006). DST was performed on Löwenstein–Jensen medium using the proportion method with the following concentrations: RIF (40 mg/L), INH (0.1, 0.2, 1, and 10 mg/L), EMB (2 mg/L), STR (4 mg/L), AMK (30 mg/L), KAN (30 mg/L), CAP (40 mg/L), OFL (2 mg/L), MOX (2 mg/L), LZD (1 mg/L), and PTH (40 mg/L) (Canetti et al., 1963; World Health Organization, 2018). PZA and BDQ were tested on 7H11 using a concentration of 300 mg/L and 0.12–0.25 mg/L, respectively, according to the proportion method (Canetti et al., 1963; World Health Organization, 2018; Heifets and Sanchez, 2000; EUCAST, 2020).

## Results

The nature of the 112 clinical specimens and the 94 strains is described in **Table 1**. The bacterial load determined by microscopic examination of the 112 clinical specimens ranged from ≥100 acid-fast bacilli (AFB) per field (*n* = 10), 10 to 99 AFB per field (*n* = 18), 1 to 9 AFB per field (*n* = 12), to <1 AFB per field but >10 on the slide (*n* = 7). Sixty-five samples were smear negative.

### Performance of GeneLEAD to Detect MTBC in Clinical Samples

Among the 112 clinical samples, 63 (56%) were detected as containing MTBC DNA by the GeneLEAD assay (**Table 2**). Fifty-eight of these positive samples grew MTBC while one grew *Mycobacterium chelonae/immunogenum* (see ^a^ on **Table 2**). Interestingly, 16 of these 58 MTBC-positive samples were smear negative. Three AFB smear-positive samples were negative by GeneLEAD and grew *Mycobacterium xenopi* (see ^b^ on **Table 2**). Finally, discrepant results were observed for four samples that yielded positive GeneLEAD results while the cultures remained negative (see ^c^ and ^d^ on **Table 2**). One of them (^c^) was weakly smear positive (<1 >10/slide), and the three others (^d^) were smear negative. These four samples were actually obtained from patients diagnosed for active TB and under treatment at time of sampling and therefore were not considered as false positive. In contrast, the sample found MTBC positive by GeneLEAD but culture positive with *M. chelonae/immunogenum* (as identified by GenoType Mycobacterium CM-AS DNA assay) was unambiguously considered as a false-positive result by GeneLEAD. Finally, it is noticeable that no inhibition of amplification was observed in the 112 clinical specimens. Taking into account all the above results, sensitivity and specificity for GeneLEAD were 100% and 98%, respectively, for the samples studied (**Table 3**).

**Table 2:** Summary of the GeneLEAD results on clinical samples.

GeneLEAD results	Clinical samples						
	Culture positive					Culture negative	
	AFB per field					AFB per field	
	≥100	10–99	1–9	<1 >10/slide	0	<1 >10/slide	0
Positive (*n* = 63)	10	17	11	5^a^	16	1^c^	3^d^
Negative (*n* = 49)	0	1^b^	1^b^	1^b^	0	0	46
Total (*n* = 112)	10	18	12	6	16	1	49

a Including one sample that grew M. chelonae/immunogenum.

b Identified as M. xenopi.

c Sample from a patient under treatment for active TB.

d Three AFB-negative samples from patients under treatment for active TB.

**Table 3:** Performances of GeneLEAD/Deeplex Myc-TB assay and microscopic examination for the detection of *M. tuberculosis* complex in 112 specimens, in comparison with culture.

MTBC culture result	MTBC identification by GeneLEAD		MTBC identification by Deeplex Myc-TB		Microscopic examination	
	Positive (*n*)	Negative (*n*)	Positive (*n*)	Negative (*n*)	Positive (*n*)	Negative (*n*)
Positive	58	0	46	12	42	16
Negative	5^a,c,d^	49^b^	0	54^a,b,c,d^	5^a,b,c^	49^d^
Sensitivity, %	100		79.3		72.4	
[95% CI]	[100–100]		[71.8–86.8]		[64.1–80.7]	
Specificity, %	98		100		90.7	
[95% CI]	[95.4–100.6]		[100–100]		[85.3–96.1]	
Cohen’s kappa	0.91		0.79		0.63	
[95% CI]	[0.86–0.96]		[0.71–0.87]		[0.54–0.72]	

a Including one sample growing M. chelonae/immunogenum.

b Including three samples growing M. xenopi.

c One sample with <1 AFB/field and >10 AFB/slide from a patient under treatment for active TB: not counted as false positive by GeneLEAD.

d Three AFB-negative samples from patients under treatment for active TB: not counted as false positive by GeneLEAD.

### Performance of GeneLEAD/Deeplex Myc-TB for Species and Spoligotype Identification

**Figure 1:**
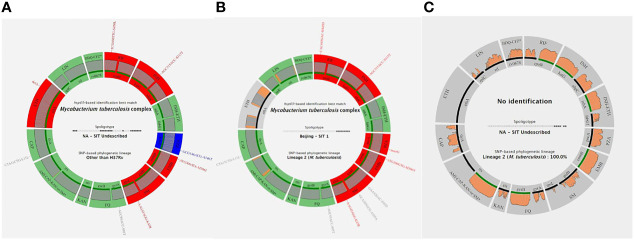
Three examples of Deeplex Myc-TB results. **(A)** Successful Deeplex Myc-TB result. **(B)** Successful Deeplex Myc-TB result with deletion in *ethA*. **(C)** Failed Deeplex Myc-TB result. *hsp65*-based identification best match, spoligotype, and SNP-based lineage identification are shown in the center of the map. Targeted loci are displayed in groups, depending on which antibiotic(s) they impact: *rpoB* for rifampicin (RIF); *katG-ahpC* for isoniazid (INH); *inhA-fabG1* for INH and ethionamide (ETH); *pncA* for pyrazinamide (PZA); *embB* for ethambutol (EMB); *rpsL-gidB* for streptomycin (SM); *gyrA-gyrB* for fluoroquinolones (FQ); *eis* for kanamycin (KAN); *rrs* for amikacin, capreomycin, and kanamycin (AMI-CAP-KAN); *tlyA* for CAP, *ethA* for ETH, *rplC-rrl* for linezolid (LNZ); and *rv0678* for bedaquiline-clofazimine (BDQ-CFZ). Wild-type alleles are shown in green, mutant alleles with mutations linked to resistance in red, and uncharacterized variants in blue (dark blue if the SNV is fixed, light blue if the SNV is unfixed). Gray color indicates that more than 5% of the locus is missing (e.g., in *ethA* in **B**). Sequencing depths are indicated above the circle representing the amplified genes, colored in gray if the read depth is >100× on average, and in orange if the read depth is <100× on average. Filled lines shown above the loci names are colored according to the amplification efficiency of each gene: green if >95% of the locus has been sequenced, and black if <95% of the locus has been sequenced. Each variant is noted with the reference codon, codon number, and the alternate codon, followed by the reference amino acid, amino acid position, and the mutated amino acid.

The results produced by the Deeplex Myc-TB assay performed on DNA extracts obtained by GeneLEAD (for clinical samples) or by heat shock (for strains) were interpreted as follows: a “successful” identification was defined as an unambiguous *hsp65*-based species identification with minimum reference coverage of 50% and minimum average coverage depth of 35×. SNP-based and spoligotype-based phylogenetic lineage determination was not taken into account when *hsp65*-based identification failed. Two examples of successful identifications are shown on **Figures 1A, B** while an unsuccessful one is shown on **Figure 1C**. Overall, the 140 successful Deeplex Myc-TB results obtained for 46 clinical samples and 94 strains yielded an average reference coverage of 93.5% and an average coverage depth of 10,190× (data not shown). As shown in **Table 3**, the Deeplex Myc-TB assay successfully identified MTBC in 46/58 (79%) of the GeneLEAD-positive samples yielding MTBC-positive cultures. On the other hand, 54 culture-negative samples remained negative by Deeplex Myc-TB. Finally, MTBC was not identified by Deeplex Myc-TB in 12 GeneLEAD-positive samples yielding MTBC-positive cultures, which were considered as false negative result by this method. Almost all of these (11/12) were obtained from smear-negative samples (*n* = 8) or samples with a weak smear positivity (<1/field >10/slide, *n* = 3). No false positive was observed by Deeplex Myc-TB testing. Based on these results, sensitivity and specificity of Deeplex Myc-TB assay performed from GeneLEAD extracted DNAs to identify MTBC were 79.3% and 100%, respectively.

Compared with the culture, sensitivity and specificity of microscopic examination performed on the 112 samples were 72.4% and 90.7%, respectively (**Table 3**).

### Performance of GeneLEAD/Deeplex Myc-TB to Detect AntiTB Drug Resistance in MTBC

The results produced by the Deeplex Myc-TB assay performed on DNA extract to detect antiTB drug resistance were evaluated on the 94 MTBC strains, and the 46 clinical specimens for which MTBC was detected by Deeplex Myc-TB and routine DST results were available.

The results yielded by Deeplex Myc-TB were concordant concerning rifampicin (RIF) for 119 (89.5%) of the 133 MTBC isolates tested (44/46 clinical culture-positive samples and 89/94 strains) (**Table 4**). With respect to the 44 clinical samples, concordance was 84% (24 RIF-R and 13 RIF-S), whereas non-synonymous uncharacterized variants (NSUVs) of *rpoB* were identified by Deeplex Myc-TB for six clinical samples (13.5%) and Deeplex Myc-TB failed (no amplification) in a smear-negative specimen (2%). With respect to the 89 MTBC strains, concordance was 92% (54 RIF-R and 28 RIF-S), while Deeplex Myc-TB found NSUVs in six strains (7%) and failed to yield result in one strain cultured in liquid MGIT (1%). Among a total of 90 *rpoB* mutations detected by Deeplex Myc-TB, the assay reported 12 (13%) NSUVs with unknown association with drug susceptibility. As shown in the **Supplementary Table 1**, the result of phenotypic DST for these 12 NSUVs was susceptible in six isolates harboring mutations M387T, D435Y, and L430P, and resistant in six others showing mutations D435Y, D435Y-Q429R, D435Y-I491L, and D435Y-S441T. Of note, two RIF-R strains carrying a rare resistance-associated mutation, S450F, encoded by a double substitution TCG450T**TC** (results confirmed by Sanger sequencing, data not shown), were reported by Deeplex Myc-TB to carry two distinct single mutations, TCG450T**T**G (99.6%) coding for the Rif-R S450L substitution, and the synonymous TCG450TC**C** (S450S) substitution (98.8%). Finally, another noticeable result was the detection by Deeplex Myc-TB of the rare V170F amino acid substitution (confirmed by Sanger sequencing) in two RIF-R strains (data not shown). Xpert and MTBDRplus DNA assays did not identify these mutations because they are located outside the rifampicin resistance determining region (RRDR) screened by these tests.

**Table 4:** Overall concordance between Deeplex Myc-TB and phenotypic DST.

Drug	Total	Concordant (culture-positive clinical samples/strains)	% (culture-positive clinical samples/strains)	Negative amplification by Deeplex Myc-TB	Discordant results or uncharacterized mutations by Deeplex Myc-TB^a^
Rifampicin	133	119 (37/82)	89.5 (84/92)	2	12
Isoniazid	133	122 (40/82)	92 (90/92)	2	9
MDR	75	69 (23/46)	92 (92/92)	0	6
Ethionamide	80	44 (16/28)	55 (64/51)	1	35^b^
Pyrazinamide	104	88 (27/61)	84.5 (93/81)	1	15
Ethambutol	130	106 (37/69)	81.5 (84/80)	1	23^c^
Fluoroquinolones	120	112 (28/84)	93 (87.5/95)	1	7
Kanamycin/amikacin	86	74 (25/49)	86 (86/86)	1	11^d^
Capreomycin	83	80 (25/55)	96.5 (96/96.5)	1	2
Streptomycin	128	94 (32/62)	73 (78/72)	1	33^e^
Linezolid	84	79 (23/56)	94 (88/96.5)	3	2^f^
Bedaquiline	83	76 (22/54)	91.5 (88/93)	1	6^g^

a Mutations detailed on **Supplementary Table 1**.

b Including one discrepancy between a -15 c->t mutation in the inhA promoter and the ETH-S DST result for one strain.

c Including 11 EMB-R-conferring mutations giving discrepant results (EMB-S) with DST (M306I, G406A or D, D354A, Q497R, S297A, and Y319S).

d Including four discrepancies between Deeplex Myc-TB and DST at the level of the eis promoter positions -10g->a (n = 1), -14c->t (n = 1), and -37g->t (n = 2), and 5 in rrs at position c1402t (n = 1), c517t (n = 2), and a906g (n = 2).

e Including 28 uncharacterized mutations.

f Two LNZ-R strains with no detectable mutations in rrl nor in rplC.

g Including two discrepancies observed in one BDQ-R strain with a frameshift not detected by Deeplex Myc-TB, and one BDQ-S strain harboring a E55D substitution in Rv0678.

Concerning isoniazid (INH), the concordance between Deeplex Myc-TB and phenotypic DST results was 92% (122/133) (**Table 4**) including 40/44 (91%) culture-positive samples (13 INH-S and 27 INH-R) and 82/89 (92%) strains (13 INH-S and 69 INH-R). Two (1.5%) Deeplex Myc-TB tests failed (no amplification for one smear-negative sample and one liquid culture) and nine discordant or uncharacterized mutations were identified (**Table 4**). Of the latter, seven corresponded to SNUVs that were all identified in INH-S strains and are detailed in **Supplementary Table 1**. Finally, no mutation was detected in two strains resistant to INH at concentrations of 0.1 and 0.2 mg/L, respectively (data not shown).

Importantly, Deeplex Myc-TB unambiguously detected resistance to both RIF and INH, i.e., multidrug resistant (MDR), in 23/25 (92%) clinical samples and 46/50 (92%) strains (**Table 4**). The six discordant results corresponded to the NSUV in *rpoB* coding for D435Y, which confers a low-level resistance to RIF and was identified either alone (two RIF-S strains), or in association with S441T (*n* = 1), Q429R (*n* = 1), and I491L (*n* = 2) in MDR strains (**Supplementary Table 1**). Thus, by interpreting the D435Y mutation as associated with RIF-R, even though the level of resistance can be low when the mutation is present alone in *rpoB*, the Deeplex Myc-TB assay was able to detect 100% of the MDR *M. tuberculosis* tested in this report.

We analyzed 80 samples with validated ethionamide (ETH) phenotypes, resulting in a poor value (55%) of agreement between Deeplex Myc-TB and DST due to 18 uncharacterized mutations, 17 divergent results, and one negative Deeplex Myc-TB PCR. The 18 NSUVs are detailed in the **Supplementary Data** and **Supplementary Table 1**. Regarding the 17 divergent results, no mutation was detected in *ethA*, or in *inhA* and its promoter for 16 ETH-R isolates (**Supplementary Table 1**). Among the remaining discrepancies, a -15 c->t substitution in the *inhA* promoter (a mutation usually involved in low-level resistance to ETH) was found in a strain susceptible to ETH (**Supplementary Table 1**).

Prediction of resistance to the two other first-line drugs pyrazinamide (PZA) and ethambutol (EMB) is summarized in **Table 4**. For PZA, 104 tests were performed on 29 clinical samples and 75 strains whose PZA DST results were available. Globally, 27/29 (93%) and 61/75 (81%) Deeplex Myc-TB results were in agreement with the phenotypic DST results, giving a concordance of 84.5% between the two methods. Deeplex Myc-TB interpreted 11 single-nucleotide variants (SNVs) as NSUVs that are detailed in **Supplementary Table 1**. For EMB, 130 samples with available DST results were analyzed, indicating a concordance of 81.5% with 106 concordant results. Apart from one PCR negative Deeplex Myc-TB test, 11 of the 23 remaining results corresponded to well-characterized EMB-R mutations giving discrepant results since they were observed in EMB-S strains: M306I (*n* = 4), G406A (*n* = 2) or D (*n* = 1), D354A (*n* = 1), Q497R (*n* = 1), S297A (*n* = 1), and Y319S (*n* = 1) (see ^c^ on **Table 4**). Twelve uncharacterized mutations were not interpreted by Deeplex Myc-TB (see details in **Supplementary Data** and **Supplementary Table 1**). Finally, one clinical strain resistant to EMB did not show mutation by Deeplex Myc-TB, or by other molecular approaches (**Supplementary Table 1**).

Of the 120 Deeplex Myc-TB tests done on samples with available DST results for fluoroquinolones (FQ), 112 were concordant with the phenotype of resistance, giving a concordance value of 93% (**Table 4**). The observed discrepancies were due to one ofloxacin (OFX) and moxifloxacin (MOX) susceptible strain harboring a A90V substitution in GyrA, which usually confers OFX-R (data not shown), six NSUVs depicted on **Supplementary Table 1**, and one Deeplex Myc-TB PCR failure.

With 94 concordant results for 128 tests, the agreement between Deeplex Myc-TB and DST for STR was 73%. Among the concordant STR-R results, RpsL substitutions K43R and K88R represented 83% of the resistance mutations. One Deeplex Myc-TB was PCR negative. Five gave rise to discrepancies with (i) two STR-S strains interpreted as STR-R by Deeplex Myc-TB because of the presence of c492t in *rrs* and a frameshift in *gidB*, respectively; (ii) one STR-R strain considered as STR-S by Deeplex Myc-TB despite the presence of a G73E substitution in GidB; and (iii) two strains without mutation in *rrs*, *gidB*, and *rpsL*, which appeared to be STR-R by DST (data not shown). Finally, we noted the presence of 28 uncharacterized mutations not interpreted by Deeplex Myc-TB that are summarized on **Supplementary Table 1** (see details in **Supplementary Data**).

The concordance value between Deeplex Myc-TB and DST determined from a set of 86 samples for injectable aminoglycosides, including KAN and AMK, was 86% (74/86, **Table 4**). We had one negative Deeplex Myc-TB PCR and 11 discordant results between Deeplex Myc-TB and DST. Nine of the 11 (see ^d^ in **Table 4**) were due to (i) four discrepancies at the level of the *eis* promoter at positions -10g->a (*n* = 1), -14 c->t (*n* = 1), and -37g->t (*n* = 2), all found in strains appearing to be both KAN-S and AMK-S, and (ii) five mutations in *rrs* at position c1402t (*n* = 1), c517t (*n* = 2), and a906g (*n* = 2) identified in KAN-S/AMK-S isolates in our study (data not shown). As shown in **Supplementary Table 1**, we also observed two NSUVs in the *eis* promoter (**Supplementary Table 1**). Finally, with 80 concordant results of 83 tests, the agreement between Deeplex Myc-TB and DST for capreomycin (CAP) is 96.5% (**Table 4**). We noticed one negative Deeplex Myc-TB amplification and two discrepancies including one uncharacterized substitution F185L in TlyA in one CAP-R strain (**Supplementary Table 1**), and one discordant result between Deeplex Myc-TB (CAP-S) and DST (CAP-R) in one strain having no mutation in the *rrs* region 1400, or in *tlyA* (data not shown).

With 79/84 Deeplex Myc-TB results in accordance with DST, the concordance value was high for linezolid (LNZ) (94%), but we noticed three Deeplex Myc-TB failures and two LNZ-R strains with no detectable mutations in *rrl* or in *rplC* (**Table 4**). We observed no uncharacterized mutations, and the only LNZ-R strain was due to the well-known substitution C154R (data not shown) (Pi et al., 2019).

As for LNZ, the concordance value for bedaquiline (BDQ) was high, 91.5% (76/83). Among the 76 concordant results, there were two BDQ-R mutants showing two missense mutations F100C and S151P in Rv0678 (data not shown). On the other hand, Deeplex Myc-TB failed in one assay and yielded two discordant results and four uncharacterized mutations (**Supplementary Table 1**). The two discordant results were due to (i) one BDQ-S strain with an E55D mutation interpreted as BDQ-R by Deeplex Myc-TB, and (ii) a wild-type strain displaying BDQ-R at low-level (R to BDQ 0.12 mg/L but S at 0.25 mg/L) (see ^g^ in **Table 4**).

### Performance of GeneLEAD/Deeplex Myc-TB to Identify MTBC Lineages and Possible Contacts Between Patients

The performance of the GeneLEAD/Deeplex Myc-TB assay for MTBC genotyping was compared to the MIRU-VNTR data that were available for 134 of the 140 clinical culture-positive samples and strains (**Supplementary Table 2**). Globally, MTB genotyping by the Deeplex Myc-TB assay from cultured strains identified the SIT number in 87% of the tests, and in 66% from clinical samples (data not shown). According to our results, a fairly good agreement between Deeplex Myc-TB spoligotyping and MIRU-VNTR was observed in lineages L1 (71% of agreement), L3 (75%), L4 LAM (72%), Haarlem (88%), X, Cameroon, and TUR (100% each). However, the concordance value dramatically dropped in L4 Ghana (42%) and in L2 Beijing (57%). In the latter clade, Deeplex Myc-TB spoligotyping, which is known to be a low discrimination approach as previously reported (Liu et al., 2014), classified 33 strains as SIT 1 while MIRU-VNTR classified them into 19 distinct patterns (**Supplementary Table 2**). Similarly, five isolates of SIT 42 (LAM9) corresponded to four distinct MIRU Mtbc15-9 codes, while nine isolates of SIT 53 were linked to eight different MIRU Mtbc15-9 codes, which were related to two clades (Ghana for eight of them and NEW for one strain); this low discriminatory power was also reported in these sublineages (Wondale et al., 2020). No epidemiological link was found within six Deeplex Myc-TB clusters representing a total of 53 strains related to SIT 1 (*n* = 31), SIT 265 (*n* = 5), SIT 42 (*n* = 5), SIT 20 (*n* = 2), SIT 53 (*n* = 8), and SIT 262 (*n* = 2). By comparison, MIRU-VNTR typing suggested eight clusters of epidemiologically unrelated strains for a total of 32 strains, e.g., for Mtbc15-9 clonal complexes 100-32 (*n* = 14), 94-32 (*n* = 4), and six other clusters gathering two to three strains (**Supplementary Table 2**). Notably, MIRU-VNTR typing and spoligotyping identified the five cases of two patients with bacteriologically and epidemiologically proven transmission. A detailed description of genotype results for the other clades is available in the **Supplementary data**.

### Time to Results and Cost of the GeneLEAD/Deeplex Myc-TB Approach

Median time from GeneLEAD clinical sample or strain reception to Deeplex Myc-TB and Sanger reporting was 8 days (IQR 7–13; 95 recorded) and 18.7 days (IQR 1–59; 73 recorded), respectively. Per strain, the cost of one Deeplex Myc-TB test (including DNA extraction with GeneLEAD, PCR, libraries preparation, and sequencing) was 1.5-fold cheaper than a complete Sanger sequencing.

## Discussion

In this prospective study, we assessed the performance of GeneLEAD combined to Deeplex Myc-TB test for identifying MTBC, predicting antiTB drug susceptibility and MTBC genotyping. GeneLEAD platform as DNA extractor has been chosen based on its theoretical high performances to produce a DNA of high quality directly from clinical samples, and to identify automatically from the extracted DNA the presence of MTBC by real-time PCR.

### Performance of GeneLEAD/Deeplex Myc-TB to Detect MTBC in Positive Samples

#### GeneLEAD Results

To our knowledge, our report is the first evaluation of the GeneLEAD system published in the literature. With 100% of the MTBC-positive cultures detected (*n* = 58), our results confirm that GeneLEAD VIII is a very promising platform for the diagnosis of TB from clinical samples, although a study with a larger sample size is needed to determine the performance of the system more accurately. It is very interesting to note that 16 (28%) out of the 58 samples that grew MTBC were smear negative, suggesting that GeneLEAD could be sensitive enough to yield detectable amounts of DNA from paucibacillary samples. No false-negative result and only one false-positive result (GeneLEAD positive–culture positive with *M. chelonae/immunogenum*) was detected, leading to a sensitivity of 100% and a specificity of 98% in the collection of the 112 samples tested. One has to note that the GeneLEAD false positive result was circumvented when the extracted DNA was further analyzed by Deeplex, which identified *M. chelonae/immunogenum*.

#### GeneLEAD/Deeplex Myc-TB Results

The Deeplex Myc-TB assay applied to GeneLEAD extracted DNA displayed performances slightly lower than the GeneLEAD alone for detecting MTBC. The sensitivity of the coupled assay was 79.3% *vs.* 100% for GeneLEAD (**Table 3**) because of 12/58 false-negative PCR carried out from MTBC culture-positive samples. All the false-negative GeneLEAD/Deeplex Myc-TB results, but one, were observed in either AFB negative samples (*n* = 8) or paucibacillary samples (<1 AFB/field and >10 AFB/slide; *n* = 3). However, one respiratory sample with 1–9 AFB/field gave a false-negative result by GeneLEAD/Deeplex Myc-TB assay. No false-positive result was obtained by coupling GeneLEAD and Deeplex Myc-TB, due to the ability of Deeplex Myc-TB to identify mycobacterial species (MTBC and >100 non-tuberculous mycobacterial species). With a sensitivity of 79.3% and a specificity of 100%, the coupled GeneLEAD/Deeplex Myc-TB assay showed encouraging performances. Compared to published results obtained with other molecular assays such as MTBDRplus and Xpert, the GeneLEAD/Deeplex Myc-TB assay could be more specific but less sensitive (Horne et al., 2019).

### Performance of GeneLEAD/Deeplex Myc-TB to Detect AntiTB Drug Resistance in MTBC

The average coverage depth that can be achieved with Deeplex Myc-TB is by far greater than that reached with WGS by NGS, whether from culture or sample, due to initial PCR amplification (Dohál et al., 2020). In our study, the 140 successful Deeplex Myc-TB results obtained for 46 clinical specimens and 94 cultured strains yielded a reference coverage of 93.5% and an average coverage depth of 10,190×, thus ensuring the user to obtain high-confidence mutation calls, including those of minor subpopulations in the case of heteroresistance. The success rate was 97.4% (37/38) for samples displaying at least 1–9 AFB/field on microscopy examination. By contrast, considering the 63 GeneLEAD-positive samples obtained from clinical samples, Deeplex Myc-TB failed in 17 cases (27%), mostly in smear-negative samples.

### Concordance

To facilitate the evaluation of Deeplex Myc-TB, we have added to the clinical samples a selection of clinical MTBC strains from the Myc-NRC representing a wide diversity of resistance traits to first- and second-line antiTB drugs. When successful, 85.4% of susceptibility predictions yielded by Deeplex Myc-TB were concordant with the results of phenotypic DST. As shown in **Table 4**, we observed a high degree of consistency between the Deeplex Myc-TB results and the DST results for RIF, INH, FQ, CAP, LNZ, and BDQ, which allows to detect MDR strains and to determine the susceptibility of the three drugs that have to be prioritized for longer MDR-TB regimens, according to WHO, namely, FQ, LNZ, and BDQ (World Health Organization, 2018). Since the results obtained for RIF and INH appeared to be robust, we were expecting to have very good results for MDR identification by Deeplex Myc-TB as well. Indeed, the rough concordance value worked out from non-interpreted data was excellent (92%). After interpreting the data by assuming that a clinical isolate is MDR when RpoB-D435Y is detected, even though it is associated with a low-level resistance to RIF, we finally concluded that all MDR strains (*n* = 75) were identified by the Deeplex Myc-TB test.

The concordance values determined for PZA, EMB, and KAN/AMK were somewhat lower than those discussed above, and were poor for STR and ETH. Such differences were mostly due to two points: (i) the proportion of uncharacterized mutations detected by Deeplex Myc-TB for a given resistance mechanism and (ii) the robustness of the susceptibility/resistance determination by DST for a given antiTB drug. Considering drugs such as RIF, INH, and FQ, the mechanisms of resistance to these drugs are extensively documented and resistance is known to stem from a limited number of very well-documented SNVs located in well-defined short stretches of the resistance genes. By contrast, for drugs such as PZA, STR, and ETH, the resistance can occur by acquisition of a wide range of mutations that can affect any position on the resistance gene plus the upstream promoter regions. For this category of drugs, the interpretations of NSUVs can be a tricky process hampering the reliability of the interpreted genotypic antibiogram.

### NSUVs

For RIF, the proportion of NSUVs related to the total number of mutations detected by Deeplex Myc-TB was 12.8%. Most of the discordant predictions were due to low-level resistance mutations poorly detected by phenotypic DST when only critical concentrations are tested, which are frequently identified in susceptible strains and resistant ones as well. Here, the substitution RpoB-D435Y was detected in two RIF-S and six RIF-R strains (**Supplementary Table 1**), and L430P was observed in two RIF-S strains even if the mutation was previously reported to be associated to low-level resistance to RIF (Deun et al., 2013; Miotto et al., 2018). From the therapeutic point of view, these two mutations decrease the RIF activity and can be associated with treatment failure using standard regimens that include 600 mg of RIF daily even if they can occur in strains detected as susceptible by DST (Williamson et al., 2012). Compared to other commercial and in-house molecular assays, Deeplex Myc-TB can detect mutations that other tests are unable to detect. With respect to RIF, molecular assays, such as the DNA strip assay MTBDRplus (Hain) or in-house PCR-sequencing of the rifampicin-resistance determining region (RRDR) of *rpoB*, cannot detect rare mutations outside the RRDR (Zaw et al., 2018). By contrast, Deeplex Myc-TB, due to its design, allows to identify any mutation outside the RRDR. In our study, we detected M387T in two Rif-S strains, and S450F and V170F in four Rif-R strains.

For INH, NSUVs represented only 9.5% of the SNVs found in *katG*, *inhA* and promoter, and *ahpC* and promoter. A striking point when compared to RIF is that all the NSUVs detected by Deeplex Myc-TB were found in INH-S strains (**Supplementary Table 1**). However, it should be noted here that several of them were detected as minority variants. Other NSUVs were phylogenetic markers, such as -142g->a in *ahpC*, which was detected in three EAI isolates, and is a phylogenetic marker of the EAI family strains. It is very likely that it is not involved in INH-R.

By contrast to INH, all but two NSUVs implicated in ETH resistance were observed in ETH-R strains (**Supplementary Table 1**). They represented 52% of the total number of SNVs found in *ethA*. All eight missense NSUVs found in EthA exhibited resistance-associated mutation characteristics as they primarily affected charged or polar residues (R207C, R239L, Q246R, S266R, and N379D) or, alternatively, introduced a proline or a STOP codon into the EthA protein (L272P, L440P, and Q459STOP) (Meehan et al., 2019). Frameshift mutations are also frequent. In our strain series, eight insertions or deletions leading to frameshifts in EthA were observed in ETH-R strains. It would be tempting here to advise Deeplex Myc-TB users to take into account the nature of the amino acid substitutions to give a phenotype interpreted from NSUVs. However, the physicochemical rules driving the effect of amino acid substitutions in proteins are too complex to consider that such an approach can give reliable results. If applied, confirmation by DST of the interpreted phenotype is mandatory to validate the genotypic interpretation.

For PZA, a very similar situation is encountered. In the *pncA* gene, 24.5% of the SNVs were NSUVs. A critical point with these mutations is to correctly interpret their effects at the level of the structure/function relationships of PncA because of the wide range of diversity of the mutations that can confer PZA-R, and also because a significant proportion of PZA-S clinical isolates display neutral PncA amino acid substitutions that have no significant effect on the pyrazinamidase activity of the enzyme and, so, on PZA susceptibility (Petrella et al., 2011).

The results obtained for EMB were harder to decipher. With 18% of NSUVs among the total number of SNVs, the discrepancies between Deeplex Myc-TB and phenotypic results were not only due to the presence of 12 NSUVs (**Supplementary Table 1**) but also due to the identification of 11 well-known EMB-R-conferring mutations giving rise to discrepant results (EMB-S) with DST (**Table 4**). One explanation for these results could be the preparation of the medium causing degradation of EMB due to the heating procedure when the DST is carried out with the Löwenstein–Jensen medium. In addition, the critical concentration chosen for the DST of EMB in our study was based on the recommendations of the proportion method (European Centre for Disease Prevention and Control, 2018), i.e., 2 mg/L of EMB. One cannot exclude that this concentration is too low compared to other reference DST methods and could contribute to the obtention of false-susceptible mutants (Schön et al., 2017). Nevertheless, four unique NSUVs not interpreted by Deeplex Myc-TB (M306L, Y334H, N296H, and Q445R) were observed in EMB-R strains. Therefore, interpreting these mutations as EMB-R, as previously suggested (Brossier et al., 2015), could significantly improve the predictiveness of EMB-R by Deeplex Myc-TB.

NSUVs represented 21% of the SNVs present in *gyrA* and *gyrB*, with only one in *gyrA* and 5 in *gyrB* (**Supplementary Table 1**). H70R in *gyrA* is an uncommon mutation previously described in a strain resistant to levofloxacin in China (Yin and Yu, 2010). With respect to the four other unique NSUVs found in GyrB (A403S, A423V, P450S, and frameshift) in FQ-S strains, they are located far from the GyrB QRDR in agreement with the susceptibility to FQ of the corresponding strains (Maruri et al., 2012). In addition, the frameshift was detected as minority mutation (7%) and might represent a yet unselected transitory event.

For the three injectable aminoglycosides, we found very few NSUVs in the genes involved in resistance to these drugs (two in the *eis* promoter and one in *tlyA*, representing 11% of all the SNVs present in the genes *rrs*, *eis*, and *tlyA*) (**Supplementary Table 1**). Since they are seemingly infrequent in clinical isolates, they should not significantly impact the Deeplex Myc-TB results. On the other hand, it is worth to mention here that Deeplex Myc-TB analysis of *rrs* and *eis* genes in our study yielded nine discrepancies between genotype and phenotype, 44% of them being located into the *eis* promoter and 56% in *rrs* (**Table 4**). The nine mutants were observed in strains KAN-S and AMK-S and represented 39% of the KAN-R and AMK-R SNVs detected in our evaluation. Such results can be put in parallel to those described above for EMB and question the reliability of the proportion method for KAN and AMK resistance evaluation, especially for mutations at positions −10, −14, and −37 of the *eis* promoter, which are assumed to confer a low-level resistance to kanamycin (Zaunbrecher et al., 2009; Pholwat et al., 2016). Another hypothesis is that other factors, not taken into account here, could modulate the effects of KAN/AMK-R SNVs, such as *whib7* previously suggested to be implicated in modifications of the activity level of KAN (Reeves et al., 2013).

Strains resistant to LNZ and BDQ have not been investigated in previous reports on Deeplex Myc-TB (Jouet et al., 2021; Kambli et al., 2021). In our study, such strains remain scarce. For LNZ, only one SNV in *rplC* determining the well-known substitution C154R was observed in a resistant strain (Pi et al., 2019), and we did not find NSUV in the other LNZ-R strains tested. For BDQ, it has been previously reported that mutations in Rv0678 are mainly responsible for low-level resistance to BDQ rather than high-level resistance (Nguyen et al., 2018). Accordingly, six strains displayed low-level resistance to BDQ in our study (7.3% of the total number of strains tested for this drug), two with frameshift mutations, two with BDQ-R conferring SNVs (F100C and S151P), one with a NSUV (R89P), and one with no detectable mutation. On the other hand, Deeplex Myc-TB found two mutations in Rv0678 in BDQ-S strains, one with a double mutation I67T-Y92STOP with a proportion of variant of 30% (**Supplementary Table 1**), and the other with the E55D substitution (**Table 4**) which is a SNV preserving the acidic function with probably no significant impact on the efflux function of Rv0678, as previously suggested (Martinez et al., 2018). The accuracy of detection of BDQ resistance, even if it remains still scarce to date, is an important challenge for MDR patient therapeutic decision. Our results underline the importance of continuing the study of resistance mechanisms to this drug.

### Detection of Minority Subpopulations

An attractive aspect of the Deeplex Myc-TB approach is its capacity to detect minority variants (up to 3%) (Jouet et al., 2021). Indeed, if minority variants can be easily detected from strains with media containing antibiotics, e.g., by using the proportion method, they could be underrepresented by phenotypic DST when starting from samples due to the fitness cost of some resistance mutations (Gygli et al., 2017). However, although treatment outcomes have been well correlated with phenotypic DST, more clinical studies are needed to evaluate the impact of minority variants that are detected by Deeplex Myc-TB from clinical samples.

### Significant Number of Resistant Strains Without Mutations

A significant number of strains displayed phenotypic resistance that remained unexplained by the Deeplex Myc-TB assay. In particular, Deeplex Myc-TB detected no mutation in 16 ETH-R clinical strains, four PZA-R, two INH-R, two STR-R, two LNZ-R, one EMB-R, and one CAP-R (**Supplementary Table 1**). For most of the drugs, the observed resistant isolates displaying wild-type profiles can be explained by the lack of some genomic targets in the Deeplex Myc-TB assay that does not cover all the putative coding sequences previously described in drug resistance, e.g., *atpE* and *pepQ* for bedaquiline (Nguyen et al., 2018). For EMB, the PCR-sequencing spans the entire *embB* gene but not the *embB* promoter located in the *embC–embA* intergenic region. For ETH, several additional putative targets have been described in resistant strains (Vilchèze and Jacobs, 2014; Zhang et al., 2017). They are not accessible to the Deeplex Myc-TB assay and could possibly explain the high number of ETH-R/wild-type genotypic results observed for this drug. Similarly, it would be useful to consider additional targets to the existing Deeplex Myc-TB panel in order to enlarge test coverage for the newest antiTB drugs. Most importantly, *fgd1*, *fbiC*, *fbiA*, *fbiB*, and *ddn* are very interesting targets for predicting resistance to pretomanid, a very promising antiTB drug, which would be welcome in the test (Dookie et al., 2018; Hameed et al., 2018; Conradie et al., 2020).

### Performance of GeneLEAD/Deeplex Myc-TB to Identify MTBC Lineages and Possible Contacts Between Patients

Deeplex Myc-TB also provides the determination of spoligotype, which can be used to highlight potential patient-to-patient transmissions and laboratory cross-contaminations, and identify recent transmission *versus* reactivation (Kamerbeek et al., 1997; Dale et al., 2001; van der Zanden et al., 2002; Supply et al., 2006; Demay et al., 2012). Here, Deeplex Myc-TB failed to identify the SIT numbers in 19 of 134 (14%) MTBC strains for which MIRU-VNTR results were available. Some of the spoligotyping patterns have not yet been reported in the SITVIT database and correspond to orphans. Non-interpretable SIT numbers were found mainly in the S (L4), West African (L5 and L6), and EAI (L1) isolates (100%, 100%, and 29%, respectively). Similar figures were reported in other literature collections (Vanhomwegen et al., 2011; Puerto et al., 2015; Shi et al., 2018).

When the spoligotype profiles obtained by Deeplex Myc-TB were compared to those obtained by MIRU-VNTR typing, a striking difference was the number of clusters identified by each typing method, with 16 clusters of two to 16 strains, plus 85 unclustered strains with unique MIRU codes for MIRU-VNTR typing, and 10 clusters of two to 33 strains, plus 52 unrelated strains with unique SIT numbers for spoligotyping (**Supplementary Table 2**). In previous reports, it has been noted that the specific discriminatory power of spoligotyping is lower than that of MIRU-VNTR, which overestimates the transmission of TB (Scott et al., 2005). Here, we confirm the lower specificity (defined as the percentage of isolates with unique pattern) of spoligotyping compared to MIRU-VNTR (39% *versus* 63.5%, respectively), in particular within the Beijing family as previously reported (Liu et al., 2014). To circumvent this low discriminatory power, the clusters determined by spoligotyping have to be further studied by MIRU-VNTR analysis or by WGS typing, as already suggested (Oelemann et al., 2007; Rasoahanitralisoa et al., 2017; Nikolayevskyy et al., 2019). Despite these drawbacks, it is generally recognized that most newly acquired infections are not detected by traditional contact tracing (Small et al., 1994; van Deutekom et al., 1997; Pfyffer et al., 1998). Therefore, DNA typing remains potentially useful in identifying non-suspect sources of transmission. The delay between suspicion of TB and the obtention of MIRU-VNTR data (usually about 1–2 months) is a major disadvantage that could be overcome by using the spoligotyping results provided by Deeplex Myc-TB, which can be made available rapidly in the GeneLEAD/Deeplex Myc-TB strategy.

### Time to Results of the GeneLEAD/Deeplex Myc-TB Approach

In the present study performed on a limited set of selected clinical samples and strains, we demonstrated at the level of a laboratory with national responsibilities that GeneLEAD/Deeplex Myc-TB can provide results on average 10 days earlier than the classical PCR-Sanger sequencing approach (data not shown). In this situation, GeneLEAD/Deeplex Myc-TB was about 1.5 less expensive than traditional routine genotyping methods if we consider that it allows the identification in a single experiment of the species, the spoligotype, and the genotypic profile of 18 resistance genes among which some genes such as *katG* would require up to 10 PCR-sequencing reactions to be fully characterized (Brossier et al., 2009). In addition, National Multidisciplinary Consultation Meetings (MCM) are held in Myc-NRC to propose the best possible cares based on microbiological, clinical, epidemiological, and molecular data (Guglielmetti et al., 2019). After our first experience limited to several clinical cases recently reviewed in MCM, it can be estimated that the GeneLEAD/Deeplex Myc-TB approach could reduce the time to treatment optimization by approximately 5–6 weeks.

### Limitations

Despite very promising performances, the capacity of the GeneLEAD/Deeplex Myc-TB approach to modify the strategy for detecting MDR and XDR TB should be evaluated in local context, in particular the local prevalence of drug resistance, the availability of WGS capacities with low turnaround time for results, and the operational responsiveness of healthcare system. For instance, we used here a high-throughput sequencing platform that provides raw data in 3 days after receiving the multiplex PCR samples, a time frame that cannot be met by all platforms. However, it is expected that the recent development of compact, portable NGS instruments will also make it possible to clinically diagnose drug-resistant TB with a Deeplex Myc-TB-based approach in low-resource settings (Tyler et al., 2018; Cabibbe et al., 2020; Mongan et al., 2020).

In conclusion, the high performances of the GeneLEAD/Deeplex Myc-TB approach for MTBC strain diagnosis makes this combined technology very promising for routine use in TB laboratory diagnosis, surveillance, and epidemiological investigations. Deeplex Myc-TB predictions of susceptibility to antiTB drugs are highly consistent with the reference phenotypic DST, except for drugs for which determination is generally difficult, such as ETH, EMB, or STR. Although spoligotyping is not as discriminating as MIRU, particularly for lineage 2, systematic determination of genotyping allows the detection of putative cases of unsuspected transmission. The addition of targets for the most recent drugs and a better interpretation of mutations not characterized by the web application have been identified as potential sources of improvement for the Deeplex Myc-TB tool. The automated web application provided with the Deeplex Myc-TB assay allows easy interpretation of the sequencing data for a large set of well-characterized variants. However, the interpretation of the results can be more challenging for a less experienced audience, especially when uncharacterized mutations are detected, which was the case for 39 susceptible and 62 resistant strains in our study, globally representing ~10% of the total number of SNPs detected.

The impressive set of molecular sensors in a single tool would make the Deeplex Myc-TB approach appropriate for countries with a high incidence of MDR-TB, or for centralized reference laboratories where a significant number of MDR strains are received. By contrast, the GeneLEAD/Deeplex Myc-TB approach would be less useful on susceptible or mono-resistant strains that can be easily identified by a pre-screening step based on DNA strip tests such as MTBDRplus and MTBDRsl, except if an epidemiological investigation is required for documenting a patient-to-patient TB transmission.

## Data Availability Statement

The original contributions presented in the study are included in the article/**Supplementary Material**. The nucleotide sequences of the NSUVs displayed in **Table 1** of the **Supplementary Material** have been deposited at GenBank under accession numbers OK562286 to OK562339. Further inquiries can be directed to the corresponding author.

## Ethics Statement

Ethical review and approval was not required for the study on human participants in accordance with the local legislation and institutional requirements. Written informed consent for participation was not required for this study in accordance with the national legislation and the institutional requirements.

## Author Contributions

WS and IB led the project and conceived and planned the tests. WS and IB took the lead in writing the manuscript. VE contributed to high-throughput sequencing, AA to resources, WS, IB, VO and FM to investigations and JR and AA to funding acquistion. All authors contributed to the article and approved the submitted version.

## Funding

This study was supported by an annual grant from Santé Publique France.

## Conflict of Interest

The authors declare that the research was conducted in the absence of any commercial or financial relationships that could be construed as a potential conflict of interest.

## Publisher’s Note

All claims expressed in this article are solely those of the authors and do not necessarily represent those of their affiliated organizations, or those of the publisher, the editors and the reviewers. Any product that may be evaluated in this article, or claim that may be made by its manufacturer, is not guaranteed or endorsed by the publisher.
