# Supplementary material for: A Comprehensive Evaluation of GeneLEAD VIII DNA Platform Combined to Deeplex Myc-TB® Assay to Detect in 8 Days Drug Resistance to 13 Antituberculous Drugs and Transmission of Mycobacterium tuberculosis Complex Directly From Clinical Samples
Source: Front Cell Infect Microbiol. 2021 Oct 29;11:707244. doi: 10.3389/fcimb.2021.707244 (PMC8586210; doi:10.3389/fcimb.2021.707244)
Supplement: Supplementary file 1 [file DataSheet_1.docx]

Supplementary material

# Supplementary Data

**Data availability:** The datasets presented in this study can be found in GenBank. The provisional names of the accession numbers are BankIt2504067, BankIt2504069, BankIt2504070, BankIt2504071, BankIt2504072, BankIt2504073, BankIt2504074, BankIt2504077, BankIt2504079, BankIt2504081 and BankIt2504082.

**Performance of GeneLEAD/Deeplex Myc-TB to detect antiTB drug resistance in MTBC**

For RIF, D435Y amino acid (AA) substitution, known to be associated with a low level of RIF resistance (34, 35), was found in 8 strains, alone in one RIF-R and 2 RIF-S strains, or associated with another AA NSUV substitution (Q429R, I491L or S441T in 1, 2, and 2 strains, respectively), all in RIF-R strains. L430P, also reported to confer low level of RIF resistance (34, 35) was found in 2 RIF-S isolates. M387T NSUV was found alone in 2 RIF-S strains. The latter mutation was also found as minority variant (<15%) in association with S450L known to confer RIF resistance, correctly identified by Deeplex Myc-TB in 3 MDR strains (data not shown).

For INH, NSUVs were found in 7 INH-S strains (Supplementary Table 1). Deeplex Myc-TB detected S140G mutations in *katG* (100% of mutant alleles) in one strain and the double mutation W161R-G234R (7% and 92% of mutant alleles, respectively) in another strain. A complex mixture of minority mutations in proportions varying from 16 to 22% (substitutions R104Q, R146Q, G234E, V267I, G273S) was observed in one clinical sample. NSUVs were detected in *ahpC*: (a) substitution R48W with a proportion of 2% relative to the wild-type allele in one strain and (b) an intergenic g->a nucleotide substitution at position -142 upstream from the *ahpC* gene in 3 INH-S strains belonging to the EAI family (2 EAI6-BGD1, and 1 EAI3-IND) (data not shown).

For ETH, Deeplex Myc-TB detected in 17 ETH-R strains infrequent non-interpretable mutations that included 1 mutation in the promoter of *ethA* (-7 t->c), 8 insertions/deletions resulting in frameshifts in *ethA*, and 8 non-synonymous *ethA* mutations leading to amino acid substitutions in EthA (R207C, R239L, Q246R, S266R, L272P, N379D, L440P, Q459STOP) (Supplementary Table 1). One *ethA* mutation determining Q269R (minority variant at 2%) was found in one strain phenotypically susceptible to ETH (Supplementary Table 1).

For PZA, the discrepancies (N=16) were due to 15 “uncharacterized” mutations that are shown on Supplementary Table 1 and 1 negative Deeplex Myc-TB PCR from a liquid culture. The A146T and I90T PncA substitutions were found in 2 and 1 PZA-S strains, respectively. Among the 12 other mutations that were found in strains/specimens growing on solid medium at 300 mg/L of PZA, we detected T167I (n=1), V7A (n=2), I133S (n=1), S67W (n=1), P62T (n=1), D8Y (n=1), and the rare mutation STOP187W (n=1). Based on the crystal structure of PncA and the numerous studies on the structure-function analysis of PncA mutants, NSUVs V7A, D8Y, S67W and I133S are located close to the structural and/or catalytic elements of the PZA binding site (Petrella et al., 2011; Karmakar et al., 2020; Supo-Escalante et al., 2020). Accordingly, they were identified in PZA-R clinical strains in our study (Supplementary Table 1). Two other mutations were related to PZA-R: Pro62Thr affecting a proline residue and STOP187W leading to the production of an extended PncA polypeptide chain with expected consequences on pyrazinamidase activity. Finally, 2 mutations occurred in PZA-S isolates in our study: I90T and A146T. The latter was previously described to be a benign mutation with no or mild effect on the level of PZA susceptibility (Walker et al., 2015).

For EMB, regarding the 12 uncharacterized mutations not interpreted by Deeplex Myc-TB, 11 were observed in EMB-R strains (M306L (n=3), Y334H (n=4), N296H (n=2), Q445R (n=1), H70R (n=1)), and one in an EMB-S strain (D496E) (Supplementary Table 1).

For fluoroquinolones, 6 NSUVs were detected by Deeplex Myc-TB, with one GyrA substitution H70R found in a FQ resistant strain, 5 GyrB substitutions in FQ-susceptible strains: A403S (n=2), A423V, P450S and a heterologous frameshift at low frequency (7%), and 1 Deeplex Myc-TB PCR failure.

For STR, the overall concordance between Deeplex Myc-TB and DST for STR is rather low (73%) (Table 4). Unlike AMK and KAN, the discrepancies did not come from discordances between well characterized mutations and DST since 81% of the STR-R conferring mutations corresponded to typical SNVs in *rrs* and *rpsL*. Strikingly, the low concordance observed for STR came from a high proportion of uncharacterized mutations in *gidB* in which we detected 28 NSUVs (31% of the total number of SNVs) (Supplementary Table 1). Note that about half of the amino acid substitutions were found in STR-S strains (N=12), and the other half in STR-R strains. Here, analysis of the types of substitutions did not allow to propose a general rule for predicting the S/R phenotype of the strains. For instance, L79S was observed twice in STR-S strains, and 4 times in STR-R (Supplementary Table 1) and the proportion of substitutions involving proline or arginine residues is approximately the same in the two phenotypic categories. Moreover, an unknown *rrs* mutation, c735g, was detected in a STR-S isolate. Among the 27 mutations occurring in *gidB*, 12 were detected in STR-S strains and 15 in STR-R strains. It is interesting to note here that the 6 frameshifts, the 2 R47P and the 6 L79S substitutions were distributed between both phenotypes STR-S and STR-R (Supplementary Table 1). For KAN and AMK, we also observed 2 uncharacterized mutations in the *eis* promoter: c>g at position -15 in a strain susceptible to both KAN and AMK, and a deletion of -8c in a strain resistant to KAN but AMK-S, and finally, for CAP, one NSUV F185L in TlyA in one CAP-R strain (Supplementary Table 1).

For BDQ, the 4 uncharacterized mutations included (i) one heterologous double mutation I67T-Y92STOP in a susceptible strain, with the nonsense mutation mixed with WT alleles in a ratio 30/70, and (ii) one R89P substitution and 2 frameshifts in 3 BDQ-R strains (Supplementary Table 1).

**Performance of GeneLEAD/Deeplex Myc-TB to identify MTBC lineages and possible contacts between patients**

Seven MTBC strains belonging to lineage 1 (EAI family) were comparatively analysed by MIRU-VNTR and by Deeplex Myc-TB after extraction on the GeneLEAD platform. As shown on Supplementary Table 2, a cluster of 2 strains sharing the same MIRU-VNTR Mtbc15-9 code, 6335-522, was also clustered by spoligotyping, with a SIT number 1340 which identified the clade EAI6-BGD1 (Supplementary Table 2). Interestingly, both strains had the same variant of ahpC (G-142A) and were isolated from patients living in the same geographical area (French Guiana and Martinique) with a delay between the two infections corresponding to patient-to-patient transmission. However, the 2 isolates were distinguishable at the level of the rpoB gene which carried 2 distinct mutations determining AA substitutions V251F and D435Y in RpoB, respectively (data not shown).

Of the 44 isolates that belonged to lineage 2, 16 were of haplotype 100-32 as defined by the MIRU-VNTR analysis. In comparison, spoligotyping by Deeplex Myc-TB assigned 12 of the 16 isolates to the SIT number 1, among which only 2 were isolated from 2 patients with bacteriologically and epidemiologically proven transmission (Supplementary Table 2). Accordingly, the 2 strains shared the same resistance genotype with AA substitutions S315T in KatG, S450L in RpoB, W68R in PncA, and N296H in EmbB (data not shown). Three other strains of haplotype 100-32 displayed unique SIT numbers, and one was not interpretable by the Deeplex Myc-TB software (Supplementary Table 2). Among the other Beijing isolates tested by Deeplex Myc-TB, 21 were also assigned to SIT1, none of them showing any epidemiologic link with the others (Supplementary Table 2). MIRU-VNTR subdivided this group of 21 strains into 3 MIRU clusters of 2 strains (1065-32, ?-32 and 19006-32) while the 15 remaining had unique MIRU Mtbc15-9 codes (Supplementary Table 2). Two strains isolated at different times in the same patient were clustered by MIRU (Mtbc15-9 = 797-32) and by Deeplex Myc-TB spoligotyping (SIT 190), as expected. Finally, 5 samples with no epidemiological link displaying a SIT number 265 were distinguished by MIRU with 4 strains of Mtbc15-9 94-32 and 1 of Mtbc15-9 1547-88 (Supplementary Table 2).

Regarding lineage 3, we analysed 4 Delhi/CAS isolates of which 3 could be spoligotyped by Deeplex Myc-TB, the last one remaining uninterpretable (Supplementary Table 2).

For lineage 4, 17 Haarlem strains were typed, forming 3 MIRU clusters of 2 strains (1319-26, 5322-15 and 190-31). Cluster 1319-26 and 5322-15 were proposed by Deeplex Myc-TB to belong to the H3 clade, with distinct SIT numbers, 99 and 50, respectively. By contrast, MIRU cluster 190-31 could not be spoligotyped by Deeplex Myc-TB (Supplementary Table 2). The 11 remaining Haarlem strains all displayed unique SIT numbers and assigned to clades H1, H3, X3 and T2, the last one being not interpretable (Supplementary Table 2).

For the 18 LAM strains, we observed 5 LAM9 strains assigned to SIT 42, which finally turned out by MIRU typing to be unique strains for 3 of them, and to belong to the same Mtbc15-9 ?-51 for 2 of them, without any link between them. Two other strains with the same Mtbc15-9 (16296-54) could not be spoligoted by Deeplex Myc-TB, while the 2 LAM1 strains from SIT 20 were clearly differentiated by MIRU (255-56 and 17828-54, respectively). Finally, of the 9 remaining strains not grouped by MIRU, 4 could be attributed to LAM9 and LAM-ZWE, but 5 remained undetermined by spoligotyping (Supplementary Table 2).

The 6 Cameroon, 12 Ghana and 2 UgandaI strains were mostly strains that were not clustered by MIRU-VNTR analysis (Supplementary Table 2). For the 6 Cameroon, a unique SIT number was attributed to each strain. In the Ghana 67-25 cluster of 3 strains, one was not interpretable by spoligotyping. Six Ghana strains linked by spoligotyping to clade T1-SIT 53 were all differentiated by MIRU-VNTR, each strain having a unique Mtbc15-9. Finally, the 2 UgandaI strains with distinct MIRU codes were assigned to group T1-171 for one strain, and not typed by Deeplex Myc-TB for the other, respectively (Supplementary Table 2).

Among the remaining L4 strains, one can note that the 4 unrelated strains of the S clade and 2/4 strains of the URAL clade could not be fully spoligotyped by Deeplex Myc-TB (Supplementary Table 2). Conversely, a SIT number was attributed by Deeplex Myc-TB to 4 TUR strains, 2/4 URAL, 2 X, 1 NEW and 5 strains linked to L4 without specific assignment to known MIRU haplotypes (Supplementary Table 2).

Finally, Deeplex Myc-TB spoligotyping identified the 3 West-African strains only at the level of the lineage (1 lineage 5 and 2 lineage 6) (Supplementary Table 2), as for the M. bovis strain which was linked by Deeplex Myc-TB to the clade Bovis (Supplementary Table 2).

# Supplementary References

1. Karmakar, M., Rodrigues, C.H.M., Horan, K., Denholm, J.T., Ascher, D.B. (2020). Structure guided prediction of Pyrazinamide resistance mutations in pncA. Sci. Rep. 10(1):1875. doi: 10.1038/s41598-020-58635-x

2. Supo-Escalante, R.R., Médico, A., Gushiken, E., Olivos-Ramírez, G.E., Quispe, Y., Torres, F., et al. (2020). Prediction of *Mycobacterium tuberculosis* pyrazinamidase function based on structural stability, physicochemical and geometrical descriptors. PloS One. 15(7):e0235643. doi: 10.1371/journal.pone.0235643

# Supplementary Figures and Tables

**Table 1. Non-synonymous uncharacterized variants (NSUV) with unknown association with drug sensitivity or resistance detected in the set of 46 clinical specimens and 94 cultured strains**

| **Drug** | **Target gene** | **Phenotypic method** | | | | |
| --- | --- | --- | --- | --- | --- | --- |
|  |  | Susceptible | N | Resistant | N |  |
| rifampicin | *rpoB* | M387T (5)^1^ | 2 | D435Y | 1 |  |
|  |  | D435Y | 2 | D435Y - Q429R | 1 |  |
|  |  | L430P | 2 | D435Y - I491L | 2 |  |
|  |  |  |  | D435Y - S441T | 2 |  |
|  |  |  |  |  |  |  |
| isoniazid | *katG* | S140G | 1 |  |  |  |
|  |  | W161R (7) - G234R (92) | 1 |  |  |  |
|  |  | Complex mix of minority mutations^2^ | 1 |  |  |  |
|  | *ahpC* | -142g->a^3^ | 3^4^ |  |  |  |
|  |  | R48W (2) | 1 |  |  |  |
|  |  |  |  |  |  |  |
| ethionamide | *ethA* | Q269R (2) | 1 | R207C | 1 |  |
|  |  |  |  | R239L | 1 |  |
|  |  |  |  | Q246R | 1 |  |
|  |  |  |  | S266R | 1 |  |
|  |  |  |  | L272P | 1 |  |
|  |  |  |  | N379D | 1 |  |
|  |  |  |  | L440P | 1 |  |
|  |  |  |  | Q459STOP | 1 |  |
|  |  |  |  | -7 t->c (*ethA*_promo_) | 1 |  |
|  |  |  |  | Frameshifts | 8 |  |
|  |  |  |  | No mutation detected | 16 |  |
|  | *inhA* _promoter_ | -15 c->t | 1 |  |  |  |
|  |  |  |  |  |  |  |
| pyrazinamide | *pncA* | A146T | 2 | T167I | 1 |  |
|  |  | I90T | 1 | V7A | 2 |  |
|  |  |  |  | I133S | 1 |  |
|  |  |  |  | S67W | 1 |  |
|  |  |  |  | P62T | 1 |  |
|  |  |  |  | D8Y | 1 |  |
|  |  |  |  | STOP187W | 1 |  |
|  |  |  |  | No mutation detected | 4 |  |
|  |  |  |  |  |  |  |
| ethambutol | *embB* | D496E | 1 | M306L | 3 |  |
|  |  |  |  | Y334H | 4 |  |
|  |  |  |  | N296H | 2 |  |
|  |  |  |  | Q445R | 1 |  |
|  |  |  |  | No mutation detected | 1 |  |
|  |  |  |  |  |  |  |
| fluoroquinolones | *gyrA* |  |  | H70R | 1 |  |
|  |  |  |  |  |  |  |
|  | *gyrB* | A403S | 2 |  |  |  |
|  |  | A423V | 1 |  |  |  |
|  |  | P450S | 1 |  |  |  |
|  |  | Frameshift (7%) | 1 |  |  |  |
|  |  |  |  |  |  |  |
| Kanamycin/amikacin | *eis* | -15 c->g | 1 | Deletion -8 c^5^ | 1 |  |
|  |  |  |  |  |  |  |
| capreomycin | *tlyA* |  |  | F185L | 1 |  |
|  |  |  |  | No mutation detected | 1 |  |
|  |  |  |  |  |  |  |
| streptomycin | *rrs* | c735g | 1 |  |  |  |
|  |  |  |  |  |  |  |
|  | *gidB* | A161D | 1 |  |  |  |
|  |  | Frameshift | 3 | Frameshift | 3 |  |
|  |  | L16R | 1 | D67H | 1 |  |
|  |  | G34V | 1 | P75S | 1 |  |
|  |  | R47P | 1 | R47P | 1 |  |
|  |  | L79S | 2 | L79S | 4 |  |
|  |  | S122P | 1 | V115G | 1 |  |
|  |  | V65A – P155S | 1 | A119D | 1 |  |
|  |  | R187W | 1 | A138P | 1 |  |
|  |  |  |  | L16R – G157R | 1 |  |
|  |  |  |  | V202M | 1 |  |
|  |  |  |  |  |  |  |
| bedaquiline | Rv0678 | I67T - Y92stop^6^ | 1 | R89P | 1 |  |
|  |  |  |  | Frameshift | 2 |  |

^1^ In parenthesis: proportion of variant when lower than 80%

^2^ For example, R104Q, R146Q, G234E, V267I, and G273S with 16-22% of mutant allele for each mutation

^3^ lower-case characters represent nucleotides, upper-case letters amino acids

^4^ In 3 EAI strains (lineage 1)

^5^ strain susceptible to AMK

^6^ heterologous mutation Y92 mixed with wild-type allele in a ratio 30/70

**Table 2. Comparison of MIRU-VNTR, Deeplex Myc-TB spoligotyping and epidemiological results for 134 samples**

| Lineage |  | MIRU-VNTR | | | | | |  | | | Deeplex Myc-TB spoligotyping | | | | | | | Epidemiological link | |  |
| --- | --- | --- | --- | --- | --- | --- | --- | --- | --- | --- | --- | --- | --- | --- | --- | --- | --- | --- | --- | --- |
|  |  | Clade | | Mtbc15-9 | | | N |  | | Clade | | | SIT number | | N | | | |  |  |
| L1 |  | | EAI | | 6335-522 | 2 | | |  | | | EAI6-BGD1 | | 1340 | | 2 | Possibly linked, but strains distinguishable by 2 distinct *rpoB* mutations | | | |
|  |  | |  | | UC^a^ | 5 | | |  | | | EAI1-SOM, EAI2, EAI3-IND | | U SITs^a^ | | 3 | No link | | | |
|  |  | |  | |  |  | | |  | | |  | | NI^b^ | | 2 |  | | | |
| L2 |  | | Beijing | | 100-32 | 16 | | |  | | | Beijing | | 1 | | 12 | Bacteriologically and epidemiologically proven transmission between 2/12 patients | | | |
|  |  | |  | |  |  | | |  | | |  | | U SITs | | 3 | No link | | | |
|  |  | |  | |  |  | | |  | | |  | | NI | | 1 |  | | | |
|  |  | |  | | 1065-32 | 2 | | |  | | | Beijing | | 1 | | 2 | No link | | | |
|  |  | |  | | ?-32 | 2 | | |  | | |  | | 1 | | 2 | No link | | | |
|  |  | |  | | 19006-32 | 2 | | |  | | |  | | 1 | | 2 | No link | | | |
|  |  | |  | | UC | 15 | | |  | | |  | | 1 | | 15 | No link | | | |
|  |  | |  | | 797-32 | 2 | | |  | | |  | | 190 | | 2 | 2 specimens from same patient | | | |
|  |  | |  | | 94-32 | 4 | | |  | | |  | | 265 | | 4 | No link | | | |
|  |  | |  | | 1547-88 | 1 | | |  | | |  | | 265 | | 1 | No link | | | |
| L3 |  | | Delhi/CAS | | UC | 4 | | |  | | | CAS1-Delhi, CAS1-Kili | | U SITs | | 3 | No link | | | |
|  |  | |  | |  |  | | |  | | | NI | | NI | | 1 |  | | | |
| L4 |  | | Haarlem | | 1319-26 | 2 | | |  | | | H3 | | 99 | | 2 | Bacteriologically and epidemiologically proven transmission between 2 patients | | | |
|  |  | |  | | 5322-15 | 2 | | |  | | | H3 | | 50 | | 2 | Bacteriologically and epidemiologically proven transmission between 2 patients | | | |
|  |  | |  | | 190-31 | 2 | | |  | | | NI | | NI | | 2 | Bacteriologically and epidemiologically proven transmission between 2 patients | | | |
|  |  | |  | | UC | 11 | | |  | | | H1 (2), H3 (3), X3 (1), T2 (1), NI (4)^c^ | | U SITs | | 11 | No link | | | |
| L4 |  | | LAM | | ?-51 | 2 | | |  | | | LAM9 | | 42 | | 2 | No link | | | |
|  |  | |  | | UC | 3 | | |  | | | LAM9 | | 42 | | 3 | No link | | | |
|  |  | |  | | 16296-54 | 2 | | |  | | | NI | | NI | | 2 | Each strain possibly linked to 2 other patients | | | |
|  |  | |  | | 255-56 / 17828-54 | 2 | | |  | | | LAM1 | | 20 | | 2 | No link | | | |
|  |  | |  | | UC | 9 | | |  | | | LAM9 (3), LAM-ZWE (1), NI (5) | | U SITs | | 9 | No link | | | |
| L4 |  | | Cameroon | | UC | 6 | | |  | | | T1 (1), T4 (1), LAM10-CAM (1), NI (3) | | U SITs | | 6 | No link | | | |
| L4 |  | | Ghana | | 67-25 | 3 | | |  | | | T1 | | 53 | | 2 | No link | | | |
|  |  | |  | |  |  | | |  | | | NI | | NI | | 1 |  | | | |
|  |  | |  | | UC | 6 | | |  | | | T1 | | 53 | | 6 | No link | | | |
|  |  | |  | | UC | 3 | | |  | | | H3 (1), X2 (1), NI (1) | | U SITs | | 3 | No link | | | |
| L4 |  | | UgandaI | | UC | 2 | | |  | | | T1 | | 171 | | 1 | No link | | | |
|  |  | |  | |  |  | | |  | | | NI | | NI | | 1 | No link | | | |
| L4 |  | | S | | UC | 4 | | |  | | | NI | | NI | | 4 | No link | | | |
| L4 |  | | TUR | | UC | 4 | | |  | | | LAM7-TUR (2), T1 (1), NI (1) | | U SITs | | 4 | No link | | | |
| L4 |  | | URAL | | 163-15 | 3 | | |  | | | H3 | | 262 | | 2 | No link | | | |
|  |  | |  | |  |  | | |  | | | NI | | NI | | 1 |  | | | |
|  |  | |  | | ?-164 | 1 | | |  | | | NI | | 774 | | 1 | No link | | | |
| L4 |  | | X | | UC | 2 | | |  | | | T1, X3 | | U SITs | | 2 | No link | | | |
| L4 |  | | NEW | | 5840 | 1 | | |  | | | T1 | | 53 | | 1 | No Link | | | |
| L4 |  | | L4 | | UC | 5 | | |  | | | T1 (2), H2 (1), LAM-9 (1), NI (1) | | U SITs | | 5 | No link | | | |
| L5 |  | | West-African 1 | | ?-21 | 1 | | |  | | | NI | | NI | | 1 |  | | | |
| L6 |  | | West-African 2 | | UC | 2 | | |  | | | NI | | NI | | 2 |  | | | |
| *M. bovis* |  | | Bovis | | 15365-149 | 1 | | |  | | | Bovis | | NI | | 1 |  | | | |

^a^ U SITs, unique SITs distinguishable from each other; UC, unclustered with unique MIRU codes

^b^ NI, not interpretable by Deeplex Myc-TB

^c^ Numbers in parenthesis indicate the number of strains for each clade
